# The Utility of Outcome Measures in Total Knee Replacement Surgery

**DOI:** 10.1155/2013/506518

**Published:** 2013-10-31

**Authors:** Michelle M. Dowsey, Peter F. M. Choong

**Affiliations:** The University of Melbourne, Department of Surgery, Saint Vincent's Hospital, 41 Victoria Parade, Fitzroy, Melbourne, VIC 3065, Australia

## Abstract

Total knee replacement (TKR) is the mainstay of treatment for people with end-stage knee OA among suitably “fit” candidates. As a high cost, high volume procedure with a worldwide demand that continues to grow it has become increasingly popular to measure response to surgery. While the majority who undergo TKR report improvements in pain and function following surgery, a significant proportion of patients report dissatisfaction with surgery as a result of ongoing pain or poor function. Poor outcomes of TKR require care that imposes on already overburdened health systems. Accurate and meaningful capture and interpretation of outcome data are imperative for appropriate patient selection, informing those at risk, and for developing strategies to mitigate the risk of poor results and dissatisfaction. The ways in which TKR outcomes are captured and analysed, the level of follow-up, the types of outcome measures used, and the timing of their application vary considerably within the literature. With this in mind, we reviewed four of the most commonly used joint specific outcome measures in TKR. We report on the utility, strengths, and limitations of the Oxford knee score (OKS), knee injury and osteoarthritis outcome score (KOOS), Western Ontario and McMaster Universities osteoarthritis index (WOMAC), and knee society clinical rating system (KSS).

## 1. Background

Total knee replacement is a major surgical procedure that requires multidisciplinary input prior to and after surgery to ensure the best possible outcome. Recovery from surgery is optimized with the inclusion of rehabilitation programs which are tailored to restore mobility and independence [1]. Time to recovery can vary following TKR, and most patients will report substantial gains between 3 and 6 months after surgery [2, 3]. Overall, a continuing pattern of improvement can be observed up to 12 months following surgery [4, 5]. While a majority of patients report improvements in pain and function following total knee replacement [6, 7], a substantial number of individuals do not meet the level of improvement expected at 12 months or more after surgery [8, 9].

A number of individual characteristics are known to influence pain and function after surgery [10]. Individual risk factors which impact on patient outcomes after TKR include age and gender [7, 11, 12], antecedent diagnosis [13], body mass index [14, 15], ethnicity [16], psychological distress [13, 17], baseline pain and functional disability [7, 13], comorbidity profile [10, 18], socioeconomic status [19], and radiographic osteoarthritis severity [7, 20]. Some of these, such as obesity and psychological distress, are potentially modifiable, making accurate and meaningful capture and interpretation of outcome data imperative for both informing those at risk and for developing strategies to mitigate the risk of poor results and dissatisfaction.

Rates of ongoing knee pain and functional impairment following TKR vary considerably in the literature, ranging from 14% to 44% of individuals reporting persistent pain [7, 9, 21, 22] and from 20% to 50% of individual was reporting functional impairment [7, 22, 23] in the first 12 to 24 months following surgery. Of note the way in which data is captured and analysed, the level of follow-up, the types of patient-reported outcome measures (PROMs) used and the timing of their application also vary considerably between these studies. Numerous instruments for measuring the outcomes of TKR exist; however, not all of them contain the necessary attributes of a “good” outcome measure. When selecting which measure to use, consideration should be given to whether the measure is appropriate for use specific to the procedure being assessed. A good outcome measure should be accessible, have demonstrated reliability and validity, place minimal burden on responders, and be responsive to change [24]. High floor and ceiling effects indicate insensitivity for detecting a change of symptoms and the maximum cut-off for floor and/or ceiling effects should be no more than 15% [24]. With this in mind, we reviewed the four most commonly used joint specific outcome measures in TKR and report on their utility, strengths, and limitations.

## 2. Oxford Knee Score (OKS)

The OKS is a knee joint specific 12-item questionnaire originally developed and validated in 1998 for use in randomised controlled trials in total knee replacement (Table 1) [25]. The OKS has 12 items, 5 for assessing pain and 7 for assessing function. Each item is worth equal weighting (1 to 5) for a total possible score ranging from 12 to 60. A lower score indicates a better outcome. The OKS is freely available at http://phi.uhce.ox.ac.uk/ox_scores.php and widely used in cohort studies and by some joint replacement registries [9, 26, 27]. A scoring manual, list of translations, and licensing information can be found via http://www.isis-innovation.com/outcomes/orthopaedic/oks.html.

The OKS is designed specifically for measuring outcomes in knee replacement. The OKS has also been used to evaluate pharmacological and conservative interventions and other knee surgery procedures in knee osteoarthritis (OA) [28]. Cross-cultural adaptations in Thai, British, Swedish, Portuguese, Dutch, German, Italian, Japanese Chinese, French, and Korean languages have been validated [29, 30]. Given the simplicity and brevity of the questionnaire, higher response rates have been reported than for other PROM's [25]; however, this is not always consistent [31].

Completion and scoring of the OKS is simple; each of 12 questions carries equal weighting (1 to 5) to provide an overall score between 12 and 60 [25]. An updated scoring method is also used, whereby each item is scored between 0 (worst outcome) and 4 (best outcome), to provide an overall score between 0 and 48 [28]. The OKS is patient administered and should take about 5 minutes to complete, and responses are based on symptoms in the preceding 4 weeks. Two missing values are accepted, and where this occurs should be replaced by the mean score for the missing item [25]. The outcome categories for the OKS have been reported based on the following cut points: excellent (>41), good (34–41), fair (27–33), and poor (<27) [32, 33]. However, these categories have not been validated and are neither commonly used nor recommended [28].

The minimum clinically important difference (MCID) estimates for the OKS as reported by Murray et al. [28] are between 3 and 5 points. These estimates are based on half the standard deviation of change in OKS scores which Murray et al. report to be is between 6 and 10 points for joint replacement studies. This interpretation is based on a systematic review of health-related quality of life instruments by Norman et al. who concluded that, in most circumstances, the threshold of discrimination for changes in health-related quality of life for chronic diseases appears to be approximately half a standard deviation of the change in outcome score [34]. In a recent study, Judge et al. reported that an 11-point or more absolute change in the OKS at 6 months after TKR discriminated the best between patients' satisfaction and a 6-month OKS ≥ 30 points identified the highest level of satisfaction [35]. A weak floor effect (7%) has been reported for the OKS prior to TKR [36]; however, ceiling effects were reported at 6 months, (14%) and 12 months, (22%) following surgery, but this was attributed to patients attaining an optimal outcome rather than a limitation of the OKS [37].

## 3. Knee Injury and Osteoarthritis Outcome Score (KOOS)

The KOOS is a knee joint specific questionnaire developed in 1998 originally for the purpose of evaluating short-term and long-term symptoms and functioning in subjects with knee injury and osteoarthritis (Table 1). It was originally validated in patients undergoing anterior cruciate ligament ACL reconstruction [38]. The KOOS is a 42-item survey designed to assess people's opinions about the difficulties they experience with activity due to problems with their knees. A higher score indicates a better outcome. The questionnaire, scoring instructions, and translations are freely available at http://www.koos.nu/. The KOOS is widely used in younger and/or more active patients with knee injury and knee osteoarthritis [39].

The KOOS has been validated for measuring outcomes in TKR [39], ACL reconstruction [38], and posttraumatic knee OA [40]. The KOOS has also been used to evaluate other OA interventions including minor knee surgery procedures [41], conservative treatments [42, 43], and nutritional [44] and pharmacological interventions [45], and population-based reference data has been published [46]. High response rates have been reported for studies of TKR in the short term: 92% at 6 months and 86% at 12 months [47]. A short-form version (KOOS-PS) which is a 7-item questionnaire derived from the original KOOS has been validated for evaluating physical function in individuals with knee OA undergoing TKR [48]. The KOOS was originally concurrently developed in English and Swedish, and numerous cross-culturally validated and translated versions exist [49]. Translations include Austrian-German, Chinese, Croatian, Czech, Danish, Dutch, Estonian, French, German, Hindi (India), Italian, Japanese, Korean, Latvian, Lithuanian, Norwegian, Persian, Polish, Portuguese, Russian, Singapore English, Slovakian, Slovenian, Spanish (Peru), Spanish (US), Thai, Turkish, and Ukrainian.

Completion of the survey is straightforward; each of the 42 items carries equal weighting (0–4). There are 5 subscales, each measuring a specific outcome: pain (9 items), symptoms (5 items), activities of daily living (17 items), sports and recreation function (5 items), and knee-related quality of life (4 items). Scores for each subscale should be calculated separately and then transformed into a score between 0 and 100 [38]. Scoring instructions and calculators are available at http://www.koos.nu/. The KOOS is patient administered and should take approximately 10–15 minutes to complete, and responses are based on symptoms in the preceding week. The process for managing missing values has been recently (2012) revised (see website), and the mean score for each subscale can be derived from a minimum response of pain (5 of 9 items), symptoms (4 of 5 items), activities of daily living (9 of 17 items), sports and recreation function (3 of 5 items), and knee-related quality of life (2 of 4 items) (http://www.koos.nu/). There are no categorical equivalents, scoring of each outcome should be reported separately, and using an aggregate score is neither recommended nor valid.

The MCID estimates for the KOOS have not been established for patients undergoing TKR. However, the minimal important change (MIC) is currently suggested to be 8–10 according to the website details, while cautioning that there are a number of patients and related factors that may impact on the MIC. Floor and ceiling effects have been reported for studies of TKR in some domains of the KOOS [39]. Preoperatively, the percentage of patients undergoing TKR with the worst possible score have reached 48% for the sports and recreation domain of the KOOS. Ceiling effects at 6 months have also been reported (15% for pain scores and 16% for sports and recreation) and at 12 months (22% for pain scores and 17% for quality of life scores).

## 4. Western Ontario and McMaster Universities Osteoarthritis Index (WOMAC)

The WOMAC was initially developed in 1982 and was first validated for the purpose of evaluating response to treatment in patients with hip and knee OA in 1998 (Table 1) [50]. The WOMAC underwent multiple subsequent revisions and refinements between 1996 and 1999 [51]. The WOMAC is a 24-item questionnaire with 3 subscales measuring pain (5 items), stiffness (2 items), and physical function (17 items). A lower score indicates a better outcome. The questionnaire, licensing information, scoring instructions, and translations are available at http://www.womac.org/.

Numerous validation studies have been conducted using the WOMAC [51]. The WOMAC has been validated for measuring outcomes in clinical trials of TKR [52] and for measuring treatment response of pharmacological interventions for knee OA [53]. It has also been used to evaluate many knee OA interventions, both surgical and conservative [54]. Response rates reaching 90% at 1 year for epidemiological studies in TKR have been reported [55, 56]. A short-form version (WOMAC-SF) which is a 7-item questionnaire derived from the physical function subscale of the WOMAC has been validated for assessing function in knee OA and TKR [57, 58]. The WOMAC is available in more than 80 languages and has been cross-culturally validated in Arabic, Chinese, Dutch, Finnish, German, Hebrew, Italian, Japanese, Korean, Moroccan, Persian, Singapore, Spanish, Swedish, Thai, and Turkish [24, 59].

Completion of the survey is straightforward; each of the 24 items has 5 possible responses for a possible score of 0 to 4 for each response. There are 3 subscales, each measuring a specific outcome: pain (5 items), stiffness (2 items), and physical function (17 items). Alternative versions are available using a visual analogue scale or numeric rating [60]. A total WOMAC score is calculated by summing the items for all 3 subscales, for a total score between 0 and 96 [61]. The WOMAC is patient administered and should take approximately 10 minutes to complete, and responses are based on symptoms in the preceding 48 hours. The method of managing missing values for the WOMAC is a variant of a standard mean imputation method. Scores of the nonmissing items for each case should be added, and the mean value is used for the missing values. However, if the patient has not replied to more than one of the 5 pain or 2 stiffness items or more than 4 of the 17 physical function items, then response for that scale is considered invalid and should not be included in the analyses [62].

The MCID estimates for the WOMAC as reported by Escobar et al. are 15 points [63]. In addition, Escobar et al. have validated the Outcome Measures in Rheumatology and Osteoarthritis Research Society International (OMERACT-OARSI) set of responder criteria in total joint replacement [52]. Patients are deemed responders or nonresponders based on a combination of absolute and relative changes of pain, function, and global patient's assessment. The criteria are as follows: (i) an improvement in pain or function ≥ 50% and an absolute change ≥ 20, then the patient is considered a responder, and (ii) if the level of improvement does not reach these criteria but improvement in at least two of the three following criteria, the patient will also be considered a responder, (a) pain ≥ 20% and absolute change ≥ 10, and (b) function ≥ 20% and absolute change ≥ 10, (c) global assessment ≥ 20% and absolute change ≥ 10.

Minimal floor effects for the WOMAC have been reported with the exception of the quality of life subscale which was reported at 14% by Roos and Toksvig-Larsen [39]. Ceiling effects have been reported for TKR at both 6 months; 27% for the pain subscale and 15% for the stiffness subscale and at 12 months; 17% for the quality of life subscale, 30% for the pain subscale, and 27% for the stiffness subscale [39, 63].

## 5. Knee Society Clinical Rating System (KSS)

The KSS is a knee joint specific questionnaire originally developed and validated in 1989 for use in assessing the outcome of total knee replacement (Table 1) [64]. The KSS has 2 components: a knee rating (0–100 points) and function (0–100 points) worth a total of 200 points. The knee rating is divided into pain (0–50 points) and a knee score which assesses range of motion, stability, and alignment (0–50 points). A higher score indicates a better outcome. The KSS is freely available at http://www.kneesociety.org/web/index.html and widely used in outcome studies for partial and total knee replacement. As a “clinician completed” scoring system aspects of its validity have been questioned by some authors [65–67]. In response to these criticisms, a revised knee society scoring system (2011-KS Score) has recently been developed [68] and validated [69] for measuring outcomes in TKR. A scoring manual, list of translations, and licensing information for both the KSS and 2011-KS can be found via http://www.kneesociety.org/web/index.html.

Despite validity issues, the KSS remains one of the most popular questionnaires amongst clinician researchers for measuring outcomes in knee replacement [68]. The KSS includes range of motion and alignment measurements, and this may in part contribute to its popularity. The importance of coronal alignment in TKR in terms of implant survival and functional outcomes has been well established in the literature [70, 71], and knee range of motion is an important marker for many activities of daily living [72]. The KSS has also been used to evaluate outcomes in other orthopaedic procedures such as high tibial osteotomy [73] and patellofemoral arthroplasty [74]. Linguistically translated versions of the KSS include Spanish [75] and Portuguese [76] and for the 2011-KS, Italian, Japanese, Mandarin Chinese, Portuguese, and Spanish. A Dutch version of the 2011-KS has also recently been validated [77]. Despite a “clinician” scoring system, the pain and function subscales of the KSS have been offered to patients to complete with high response rates reported at 12 months or more [7, 78].

Completion and scoring of the KSS are simple; the function subscale (0–100) is based on walking distance (0–50) and ability to climb stairs (0–50) with deductions for use of a gait aid (0–20). The pain subscale is (0–50) and the knee rating (0–50) is based on range of motion (0–25) and knee stability (0–25) with deductions made dependent on the existence and severity of flexion contracture (0–15), extension lag (0–15), and malalignment (0–20) [64]. A negative score is possible and should be converted to zero. A web-based calculator is available at http://www.orthoscores.com/scorepages/knee_society_score.html. Categories for the KSS have been established but not validated [79]. Cut points for each of the 2 subscales are excellent (≥80), good (70–79), fair (60–69), and poor (<60). The KSS is clinician administered and should take less than 10 minutes to complete. The pain and function subscales should take about 5 minutes to complete whether by clinician or patient, and responses are based on symptoms in the preceding 4 weeks. No instruction on managing missing items could be found.

The 2011-KS scoring manual and instructions can be requested via the knee society website above. The 2011-KS expands on the KSS and includes subscales for patient satisfaction (5 items, 0–40 points), expectation (3 items, 0–15 points), and functional activities (19 items, 0–100 points), which is divided into functional activities (5 items, 0–30 points), standard activities (6 items, 0–30 points), advanced activities (5 items, 0–25 points), and discretionary knee activities (3 items 0–15 points) [68]. Satisfaction expectation and function should be reported as separate scores as a composite score is not recommended. The suggested method for managing missing values is to enter dummy values equal to the average of all of the other items in the same domain. This should be limited to instances where fewer than 50% of responses are missing [80].

The MCID estimates for the KSS and 2011-KS have not been identified for patients undergoing TKR. However, in a study by Jacobs and Christensen, a minimum change of 34.5 points at 3 months in the function subscale of the KSS was established as clinically important [81]. Ceiling effects have been reported for studies of TKR in both the knee (25%) and function (43%) subscales of the original KSS at 12 months [82]. Floor effects did not occur preoperatively and ceiling effects did not occur at 6 months after TKR in a Dutch study validating the KS-2011 [77].

## 6. Summary

Total knee replacement remains the mainstay of treatment for people with end-stage knee OA (among suitably “fit” candidates). As a high cost, high volume procedure with a worldwide demand that continues to grow, it is becoming increasingly important to understand the drivers behind response to surgery [24]. Poor outcomes of TKR require care that is an imposition on an already overburdened health system. Not only will there be a demand for ongoing outpatient specialist and community health consultations, persistent use of prescription medication, prolonged requirement for allied health services (physiotherapy and occupational therapy), and the possible need for repeated minor (arthroscopic) and major (revision joint replacement) surgery, these activities potentially deprive or delay other patients with untreated OA from receiving expeditious care.

Numerous instruments for measuring outcomes in TKR have been developed and validated over time [24] in an attempt to capture response to surgery and predict those who may be at risk of suboptimal results. We have presented a summary of the utility, strengths, and limitations of four of the most commonly used outcome measures for total knee replacement. Generic strengths among the four outcome measures included the relatively minimal burden required to complete each instrument and their design specific to measuring TKR outcomes, whereas ceiling and/or floor effects were a limitation to varying degrees for each of the four outcome measures, with the exception of the 2011-KS which requires further validation studies. No single outcome measure would be suitable for every foreseeable clinical situation or research activity. The individual strengths of each outcome measure may be useful in guiding the decision as to which measure is best suited for use, in any given situation. We noted a number of individual strengths amongst the outcome measures presented in this review.

The OKS is freely available and noted for its simplicity and brevity and appears to be the measure of choice for large data sets and joint registry's [9, 26, 27]. The KOOS is also freely available and aside from TKR is valuable for measuring outcomes in younger and/or more active patients with knee injury and knee osteoarthritis. The KOOS is also used to measure outcomes following a range of both surgical and conservative interventions of the knee, both surgical and conservative, making it attractive for treatment comparisons [38–45]. WOMAC scores can also be derived from the KOOS. An important aspect of the utility of any outcome measure is the availability of responder definitions and cutoff points and that these are appropriately validated. The WOMAC is currently the only outcome measure that has validated responder definitions and cutoff points specifically for TKR [52]. Having a set of established and validated response criteria makes the WOMAC an excellent option for use in clinical trials that aim to measure response to TKR and other nonsurgical interventions [52, 53]. It also has the most extensive range of translations available. Despite validity issues, the KSS remains one of the most popular rating systems for measuring outcomes in TKR [68]. It is one of the few outcome measures that include assessment of clinical measures that are deemed important in terms of implant survival and functional outcomes [70, 71]. The 2011-KS also includes measures of patient expectation and satisfaction which are emerging as important adjuncts in measuring response to surgery [83].

While the utility of any one particular outcome measure over another continues to be debated and the number of available instruments continues to increase, we believe that there are 2 key factors that are essential in producing quality outcome data irrespective of the instrument used. Firstly, recording of baseline scores is essential for producing meaningful outcome data. It is well established that better baseline scores correlate with better outcome scores and those with the worst baseline scores demonstrate the greatest amount of improvement [13]. Therefore, at a minimum, data analyses should always be adjusted for baseline when either presenting outcome scores or measuring the change in scores. Finally, individuals who do not respond to surveys report significantly poorer outcomes than those who do [78]. As such establishing a process for data collection that ensures the highest possible response rate such as those used by Bourne et al. will minimise nonresponder bias [83].

## Figures and Tables

**Table 1:** Patient outcome measures in knee osteoarthritis severity.

PROM	Scoring	Response criteria	Validated for use	Translation
OKS [25] Dawson et al. 1998	12 items (pain; 5 items, function; 7 items), each item worth 0–4 points. Range 0–48 points Categories: excellent (>41), good (34–41), fair (27–33), and poor (<27)	MCID 3–5 points change in score [28] ≥30 points at 6 months after TKR [35]	TKR	Thai, British, Swedish, Dutch, Portuguese, German, Italian, Japanese, Chinese, Korean, and French

WOMAC [50] Bellamy et al. 1988	24 items (pain; 5 items, stiffness; 2 items, and physical function; 17 items) Range from 0 to 96 points.	MCID 15 points [63] Responder criteria [52]: Improvement in pain or function ≥ 50% and absolute change ≥ 20 Or 2 of the 3 improvements as follows: (i) pain ≥ 20% and absolute change ≥ 10, (ii) function ≥ 20% and absolute change ≥ 10, (iii) global assessment ≥ 20% and absolute change ≥ 10.	TKR OA Post traumatic OA	>80 languages Arabic, Chinese, Dutch, Finnish, German, Hebrew, Italian, Japanese, Korean, Moroccan, Persian, Singapore, Spanish, Swedish, Thai, and Turkish

KSS [64] Insall et al. 1989 2011-KS [68] Scuderi et al. 2012	KSS-2 components: a knee rating (0–100 points) and function (0–100 points) Categories: excellent (≥80), good (70–79), fair (60–69), and poor (<60) 2011 KSS-5 subscales: knee score (0–50), pain score (0–25), satisfaction (0–40), expectation (0–15), and function (0–100)	MCID 34.5 points improvement in functional subscale [81]	2011 KS TKR UCKR	KSS-Portuguese, Spanish 2011-KSS-Italian, Japanese, Mandarin Chinese, Dutch, Portuguese, and Spanish

KOOS [38] ROOS et al. 1998	42 items, 5 subscales: pain (9 items), symptoms (5 items), activities of daily living (17 items), sports and recreation (5 items), and quality of life (4 items). Each item worth 0–4 points. Scores for each subscale are calculated separately and then transformed into a score between 0 and 100.	MCID 8–10 point change in score	TKR ACL Reconstruction Posttraumatic OA	Austrian-German, Chinese, Croatian, Czech, Ukrainian, Estonian, French, German, Hindi (India), Italian, Thai, Japanese, Korean, Latvian, Lithuanian, Norwegian, Persian, Polish, Portuguese, Russian, Dutch, Singapore, English, Slovakian, Spanish, Slovenian, Turkish, and Danish
